# Pantothenic Acid as an Unexpected Cause of Hypersensitivity to Sensitive or Atopic Skin Care Products—A Narrative Review

**DOI:** 10.3390/pharmaceutics18070771

**Published:** 2026-06-24

**Authors:** Kinga Lis

**Affiliations:** Department of Allergology, Clinical Immunology and Internal Medicine, Ludwik Rydygier Collegium Medicum in Bydgoszcz, Nicolaus Copernicus University in Torun, ul. Ujejskiego 75, 85-168 Bydgoszcz, Poland; kinga.lis@cm.umk.pl

**Keywords:** pantothenic acid, panthenol, vitamin B5, hypersensitivity, allergy

## Abstract

Pantothenic acid (PA; vitamin B5) is an essential component of many key metabolic processes. Vitamin B5 deficiency causes dysfunction in various systems and organs. Humans do not produce vitamin B5, so it must be constantly replenished through diet or supplementation. Pantothenic acid is synthesized by plants, fungi, and bacteria, so a well-balanced diet is a good source of pantothenic acid. Pantothenates have beneficial effects on the skin (regenerative, healing, and anti-inflammatory effects). They are readily absorbed through the skin, making them a common active ingredient in cosmetics and medications with soothing, healing, regenerative, moisturizing, and protective properties for damaged, sensitive, or atopic skin, including topical products labeled hypoallergenic or specifically intended for people with sensitive skin. Although PA is considered non-allergenic and safe, paradoxically, frequent exposure, especially to damaged skin, appears to lead to hypersensitivity to this substance. The pathogenetic mechanisms of pantothenate hypersensitivity have not been clearly defined. The main sensitization pathway is likely a delayed cellular mechanism (type IV, contact). However, other types of hypersensitivity, including immediate (type I) and mixed mechanisms, cannot be ruled out. PA allergy is considered rare and therefore difficult to diagnose. This is due to the unexpected sensitizing factor and the lack of standard diagnostic tests. Due to the likely contact nature of the allergy, patch testing (PT) with a cosmetic (drug) provided by the patient (“as is”) and PA (5% in petrolatum; 5% PET) appears to be the best approach. It is also suggested that PA (5% PET>) be included in the standard series of allergens (haptens) used in routine PT diagnostics. It appears that PA allergy is more common than currently believed, particularly in people with atopic skin and polysensitized individuals, who are the primary users of products containing pantothenate. It is possible that in these individuals, pantothenic acid may be more harmful than beneficial.

## 1. Introduction

**Figure 1:**
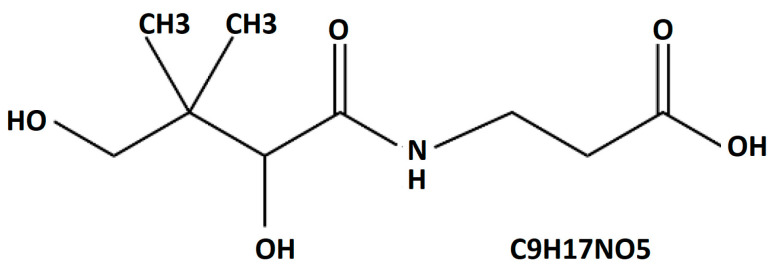
Pantothenic acid—molecular and structural formula (author’s own figure based on [5,6,7]).

Pantothenic acid, also known as vitamin B5, belongs to the group of water-soluble B vitamins [1,2]. The occurrence of pantothenic acid in nature is common, which is reflected in the name of this compound, which comes from the Greek word “pantothen” (or “panthos”), meaning “ubiquitous” [3]. In nature, the D isomer occurs naturally (Figure 1), which is the biologically active form [4].

Vitamin B5 is essential for maintaining the proper structure of the skin, hair and nails, supports hair pigmentation, supports wound healing, inhibits inflammation of the mucous membranes, participates in the process of producing acetylcholine and influences the proper growth of the body [4,8].

The regenerative and anti-inflammatory properties of vitamin B5 are used in medicines and cosmetics for various skincare and therapeutic purposes, including cosmetics and medicines that soothe irritation and inflammation and support the healing and regeneration of damaged skin [2,9,10]. Pantothenic acid is considered a safe and non-allergenic substance. Hypersensitivity to vitamin B5 is considered a very rare phenomenon. However, there are speculations that this allergy is underestimated and may be more common than currently believed [7,8].

## 2. Purpose and Method of the Review

This narrative review focuses on the analysis of hypersensitivity reactions to pantothenic acid, taking into account clinical manifestations, potential pathogenic mechanisms, and current diagnostic options. The review was conducted based on available literature published up to 12 January 2026. Data were searched using keywords (listed below) in PubMed (primarily), EMBASE, Google Scholar, and publicly available internet search engines (e.g., Google). Search results from basic internet search engines were considered only as a supplementary starting point for the medical database search. Example search terms included: hypersensitivity/allergy to vitamin B5, hypersensitivity/allergy to pantothenic acid, hypersensitivity/allergy to pantothenic acid derivatives, hypersensitivity/allergy to pantothenic acid metabolites, etc. Both review publications and published clinical cases, along with source references, were included in the review.

## 3. Pantothenic Acid

### 3.1. History of Discovery and General Information

Pantothenic acid was discovered in 1931 by Williams et al. [11] while studying the growth of *Saccharomyces cerevisiae* colonies. These researchers noticed that yeast cells secrete a specific, acidic substance that stimulated the growth of the colonies. In 1933, they gave it the name pantothenic acid from the Greek word “panthos” (or “pantothen”), which is translated as “from all sides” [3]. This name well reflected the commonness of its occurrence in food, discovered during research on vitamin B5. Interestingly, pantothenic acid practically does not occur in the natural environment in its free state. It is also difficult to obtain it by chemical synthesis [12]. In subsequent years, studies conducted on chickens [13,14] and rats [15,16] confirmed the essential role of pantothenic acid in the proper metabolism of animals and the necessity of providing this compound in everyday food. These observations were also applied to humans [17].

In 1938 Williams [18] isolated pantothenic acid from sheep liver, and in 1940 he determined the structure of this molecule [19]. In 1940 the industrial synthesis of vitamin B5 began [20].

### 3.2. Physical and Chemical Properties

Pantothenic acid consists of pantoic acid and β-alanine linked by an amide bond (Figure 1). The active, naturally occurring form of this vitamin is D-pantothenic acid [21]. Pure pantothenic acid is a water-soluble, viscous, yellow oil. The pKa of pantothenic acid is 4.41. It is stable at neutral pH but is easily degraded in both acidic and alkaline environments [4]. It is sensitive to high temperatures. Pantothenic acid salts and panthenol are less sensitive to environmental conditions and therefore are used in cosmetic, pharmaceutical and supplement preparations. The most commonly used are the calcium or sodium salt of D-pantothenic acid [4,7,20]. Calcium pantothenate and sodium pantothenate are solid substances, in the form of protein crystalline, odorless powder. Sodium pantothenate is more hygroscopic than the calcium salt of pantothenic acid, which limits its use [2,4,20]. Both salts of pantothenic acid, after being absorbed into the body, are metabolized into D-pantothenic acid, which is the active form of vitamin B5 [2,4,20].

### 3.3. Biological Functions

Pantothenic acid participates in the synthesis of coenzyme A (CoA) and acyl carrier protein (ACP). CoA is an organic chemical compound composed of 3′-phosphoryladenosine diphosphate, pantothenate, and cysteamine residues. CoA and ACP are essential for the function of many enzymes in important metabolic pathways. It is estimated that approximately 4% of cellular enzymes require CoA or its thioester derivatives for their enzymatic activity. They participate in major metabolic processes, including the Krebs cycle, a fundamental process of cellular metabolism, as well as in the synthesis of fats, steroid hormones, hemoglobin porphyrin rings, neurotransmitters, and vitamin D, and vitamin A metabolism (Table 1) [2,22,23,24,25,26].

### 3.4. Deficiency Symptoms

Due to the crucial role of vitamin B5 in maintaining the body’s metabolic and energy homeostasis, a deficiency of this vitamin causes impaired function in various systems and organs. Both somatic and behavioral disorders may occur (Table 2).

### 3.5. Sources of Pantothenic Acid and Daily Requirement

Humans, like all animals, do not produce vitamin B5 and because pantothenic acid is water-soluble, excess vitamin B5 is excreted in the urine and only small amounts are stored in the body. A positive linear correlation is observed between the intake of pantothenic acid in the amount of 4.8–6.3 mg/day and the concentration of vitamin B5 in 24-h urine. For these reasons, it requires systematic supplementation from food [2,9,28,29]. The daily requirement for vitamin B5 varies and depends on the age, general condition and clinical situation of the patient (Table 3).

Vitamin B5 biosynthesis occurs in plants and fungi. Most bacteria also have the ability to produce this vitamin. Vitamin B5 is ubiquitously present in most foods, where it occurs primarily in the form of CoA or phosphoprotein (Table 4) [4,28,36,37,38].

Additional endogenous sources of pantothenic acid in the human body include the degradation of endogenous CoA and metabolic products of the gut microbiome. However, the contribution of these two sources to the body’s overall vitamin B5 supply is small and does not meet its needs [4,26,37,38,40,41,42,43,44,45].

Pantothenic acid, primarily in the form of calcium pantothenate, sodium pantothenate, or dexpanthenol, is also a common food fortifier. Vitamin B5 is primarily added to various types of foods for special nutritional purposes, such as sports products, infant formula, low-calorie and/or reduced-calorie foods, and high-vitamin foods used in cases of malnutrition or increased vitamin requirements [4,28,29,37].

### 3.6. Local Anti-Inflammatory and Regenerative Effect

Pantothenic acid is used in topical (skin) products in the form of panthenol, a stable alcohol analogue. There are two enantiomers of panthenol, L-panthenol and D-panthenol (dexpanthenol), of which dexpanthenol is used in cosmetics and medicine as a biologically active form that penetrates the skin well [7,46,47,48,49]. After absorption into the skin, dexpanthenol is rapidly enzymatically converted to pantothenic acid, a precursor of coenzyme A. It then participates in a series of enzymatic processes crucial for maintaining the well-being of the skin and mucous membranes [50], including the synthesis of fatty acids and sphingolipids in the lipid layers of the stratum corneum [48]. Panthenol, when applied topically, supports wound healing at the cellular level by inducing fibroblast proliferation and accelerating epithelialization. Panthenol also molecularly modulates the expression of key genes involved in this process [48,51]. Panthenol rebuilds and stabilizes the function of the epidermal barrier, significantly reduces transepidermal water loss and improves the hydration of the stratum corneum [48,49,52].

The first ointment containing dexpanthenol (Bepanthen^®^) was introduced to the market in 1944. It was Bepanten Baby^®^, a protective ointment for infants, and is still available today [48,53]. Its healing, soothing, and moisturizing properties have made dexpanthenol a common ingredient in cosmetics and topical therapeutic or protective products intended for application to the skin or mucous membranes, such as ointments, creams, foams, gels, and sprays. Vitamin B5 is also a common active ingredient in essential daily skin, hair, and nail care products (e.g., moisturizing, soothing, and protective creams, shampoos and hair conditioners, and nail regenerating conditioners) [7,47,48,54,55].

## 4. Hypersensitivity to Vitamin B5

### 4.1. Clinical Aspect

Vitamin B5 is considered a relatively safe substance that is unlikely to cause hypersensitivity reactions. However, this phenomenon may be underestimated due to the fact that panthenol is a component of drugs and/or cosmetics used specifically for inflammation or irritation of the skin or mucous membranes, similarly to lanolin hypersensitivity [56,57].

**Figure 2:**
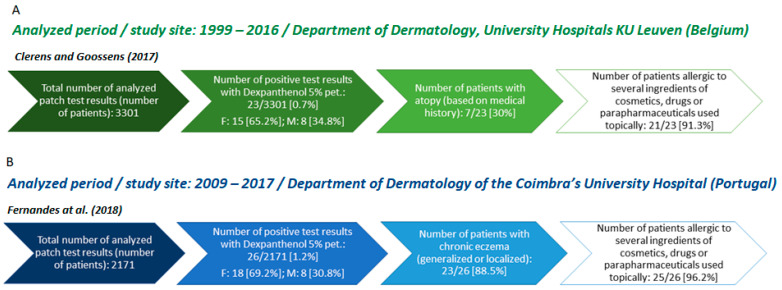
Sensitization to dexpanthenol based on the results of retrospective analysis of epidermal patch test results with basic allergen series from (**A**) the Belgian center [58] and (**B**) the Portuguese center [59] (author’s own figure).

Based on available retrospective studies [58,59], whose authors analyzed the results of epidermal patch tests performed with standard series of allergens/haptens, the frequency of positive results for dexpanthenol (dexpanthenol 5% pet.) is 0.7% in the study by Clarens and Goossens [58] and 1.2% in the study by Fernandes et al. [59] (Figure 2). In both analyses, it was also observed that dexpanthenol allergy more often affects women and people who are simultaneously allergic to other cosmetic ingredients (e.g., fragrances, emulsifiers, or other additives), people with atopy, or those using panthenol on inflamed or damaged skin (Figure 2) [58,59].

**Figure 3:**
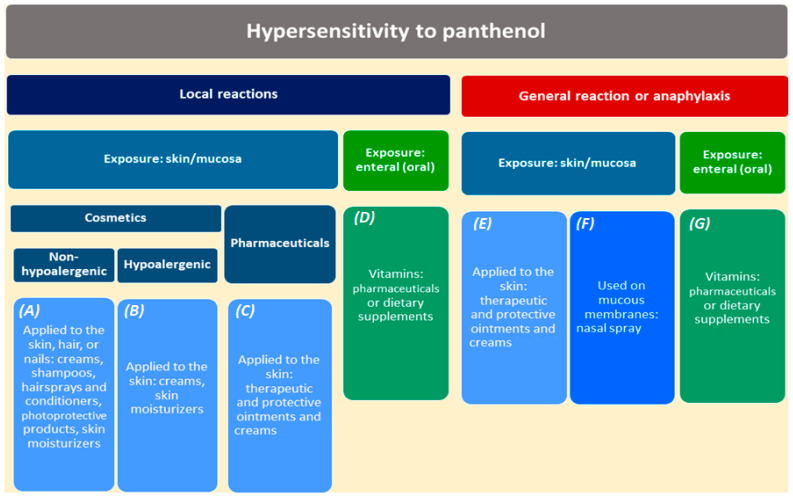
Panthenol hypersensitivity based on reported clinical cases; (**A**) [61,64,65,66,67,68], (**B**) [52,69,70]; (**C**) [71,72,73,74,75,76,77,78,79], (**D**) [62]; (**E**) [80,81], (**F**) [82], (**G**) [63] (author’s own figure).

For example, according to PubMed data [60], 25 clinical case reports of contact dermatitis caused by panthenol have been published so far, starting from 1981, when the first such case was reported [61]. Most of the reported hypersensitivity reactions to vitamin B5 were mainly caused by the local use of various cosmetics, care products, or medicinal products, including hypoallergenic preparations applied to the skin or mucous membranes (Figure 3). Only two cases of hypersensitivity reactions to panthenol after oral administration were reported [62,63].

Hypersensitivity to panthenol is primarily a delayed or late local reaction limited to the area of application of the product containing vitamin B5. Clinically, skin reactions such as redness, itching, burning, rash of various types, and swelling are observed. Urticarial blisters or exudative wounds may occur. Systemic reactions (e.g., generalized urticaria, shortness of breath, or other respiratory disorders) are very rare [8,69,80,81,82]. Allergy to panthenol may also cause reactions in areas distant from the application site, such as watery eyes or conjunctival swelling [68]. Skin lesions in areas other than those exposed to vitamin B5 are also possible [81].

### 4.2. Special Aspect—Hypoallergenic Cosmetics and Cosmetics for Atopic Skin

Based on the study by Weber and Hylwa [83], 12 clinical cases of dermatitis caused by various cosmetic or medicinal products containing panthenol, which was the cause of these hypersensitivity reactions, were published in the years 1988–2024. More than 40% of these cases occurred after using various types of cosmetics from the “sensitive”, “mild”, “natural” or “hypoallergenic” groups.

**Figure 4:**
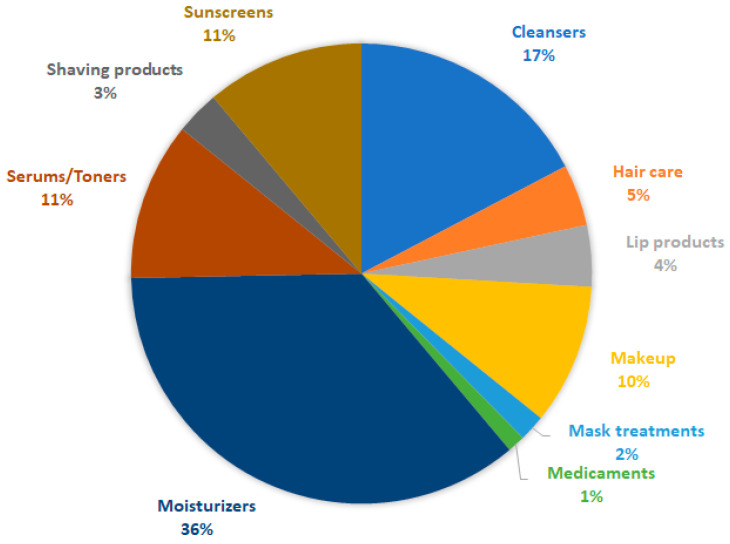
Commercially available cosmetics classified by the manufacturer as “hypoallergenic” or “for sensitive skin” based on the analysis by Weber & Hylwa (2025) [83] (author’s own figure based on [83]).

According to these authors [83], cosmetics described as “hypoallergenic”, “sensitive”, “mild” or “natural” have become an increasingly common cause of atopic contact dermatitis in recent years. Panthenol is a common active ingredient in these products. Weber and colleagues analyzed the composition of commercially available “hypoallergenic” or for “sensitive skin” cosmetics from several leading manufacturers. According to their report, in 2025, 162 cosmetics of this type containing panthenol were available for hygiene and care of various skin areas (Figure 4).

It is noteworthy that 36% were moisturizing cosmetics, which are often recommended for the care of dry, flaky, inflamed, or atopic skin [83]. This appears to increase the likelihood of panthenol hypersensitivity. It has been observed that applying panthenol to inflamed or mechanically [77] and/or chemically [75] damaged skin increases the risk of panthenol allergy. It is also possible that panthenol may cause hypersensitivity when applied to damaged skin but not cause any adverse reactions if the same person applies it to healthy skin [71,79]. Skin damage facilitates the penetration of allergens into the deep skin layer and enables quick access to immunocompetent cells, including antigen-presenting cells (APC), which predisposes to the development of sensitization [84,85,86,87]. A similar phenomenon has been observed for lanolin [57,87,88].

### 4.3. Probable Mechanism(s)

**Figure 5:**
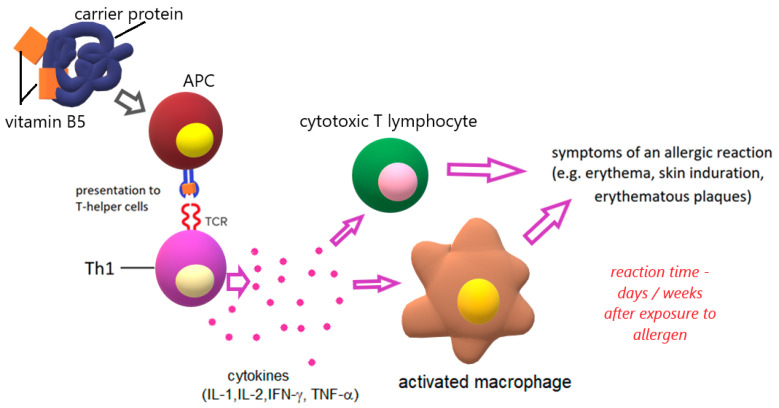
Mechanism of type IV hypersensitivity. Th1—helper lymphocyte type 1; APC—antigen-presenting cell; IL—interleukin; TNF—tumor necrosis factor; IFN—interferon; TCR—T-cell receptor (author’s own figure based on [91]).

The mechanism of hypersensitivity to panthenol has not been clearly explained. The dynamics of reaction development and the local nature of the clinical manifestations suggest that hypersensitivity to pantothenic acid and its derivatives occurs via a type IV hypersensitivity mechanism, with vitamin B5 as the hapten (Figure 5) [63,73,74,89,90].

Hahn et al. [90] observed an increase in lymphocyte proliferation in response to dexpanthenol using a lymphocyte transformation test (LTT) after pre-incubation of lymphocytes with dexpanthenol-modified microsomes compared to lymphocytes incubated with dexpanthenol without microsomes. This suggests that microsomal enzymes (such as cytochrome P450) likely play a significant role in the pathogenesis of dexpanthenol allergy. It seems likely that panthenol hypersensitivity is a specific T-cell-dependent reaction (type IV hypersensitivity; Figure 5), enhanced by microsome-dependent antigen metabolism.

The few reported cases of systemic reactions [80,81,82] including anaphylaxis [63] do not allow for clear conclusions on this subject and suggest that other pathogenetic pathways of hypersensitivity reactions to vitamin B5 are also possible.

**Figure 6:**
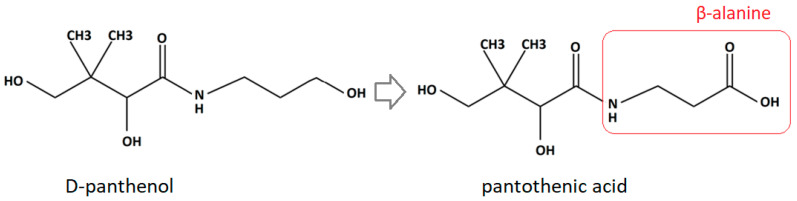
D-panthenol and pantothenic acid with the β-alanine fragment highlighted (red frame) (author’s own figure).

It is also unclear which fragment(s) of the pantothenic acid molecule or its derivatives are immunogenic. According to available literature, pantothenic acid, but not its derivatives, has immunogenic properties. However, after absorption, stable pantothenic acid derivatives are rapidly metabolized to biologically active pantothenic acid in a two-step oxidation process [7]. The β-alanine fragment of pantothenic acid is suspected to have immunogenic properties and play a major role in the pathophysiology of allergy to panthenol and its derivatives (Figure 6) [63,73,74,89,90].

The development of haptens into complete immunogens through metabolic transformation is characteristic of this group of antigens. However, vitamin B5 metabolism is multi-stage [2] and it cannot be ruled out that at any stage of endogenous pantothenate metabolism, epitopes stimulating hypersensitivity reactions may arise [90].

**Figure 7:**
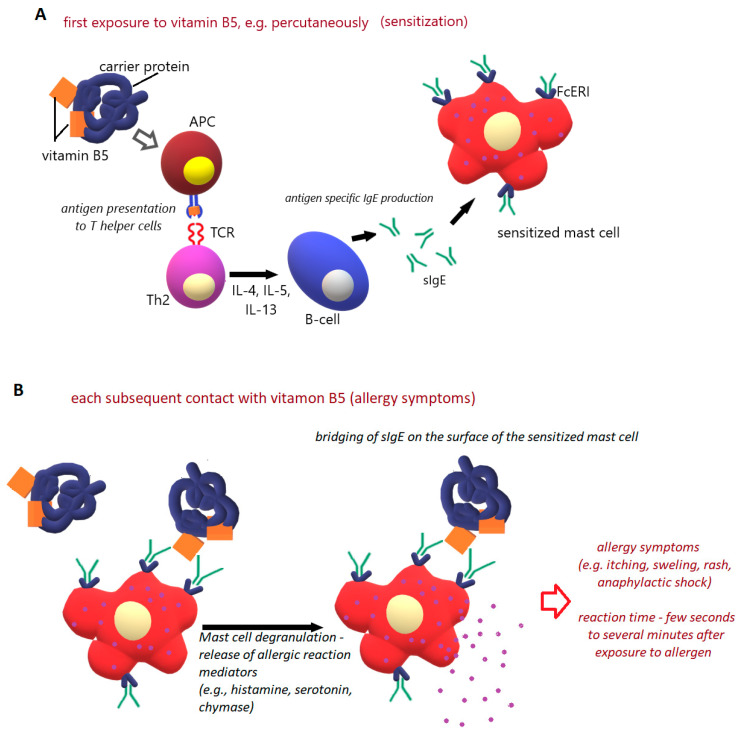
Hypersensitivity to vitamin B5 in the immediate reaction mechanism (type I); (**A**) sensitization phase; (**B**) effector phase; Th2—helper lymphocyte type 2; APC—antigen-presenting cell; TCR—T-cell receptor; IL—interleukin; FcERI—high affinity receptor for immunoglobulin E; sIgE—specific immunoglobulin E (author’s own figure based on [92]).

Rockmann et al. [63] described a case of anaphylaxis (facial swelling involving the eyelids and tongue, shortness of breath, dizziness, and fainting) that occurred after oral ingestion of a multivitamin tablet with dexpanthenol in a 30-year-old woman with a history of allergy to cosmetics containing panthenol. Reactions such as itchy lips, tongue swelling, and eyelid swelling of varying severity occurred in the patient several times in the period preceding anaphylaxis, always within a dozen or so minutes of taking the vitamins (various multivitamin preparations always containing dexpanthenol). The allergy to dexpanthenol was confirmed by scratch testing with the multivitamin tablets used by the patient (as is) and by a skin friction test with dexpanthenol (5% pet.). In both tests, the patient experienced systemic reactions requiring medical intervention. The total serum IgE concentration was low at 9.0 kU/L. Based on the clinical symptoms of the reaction, medical history, and diagnostic test results, Rockmann et al. [63] diagnosed the patient with both contact (a history of a local reaction to a sunscreen containing panthenol) and systemic (systemic symptoms after oral ingestion of dexpanthenol) allergy to dexpanthenol. They did not determine the mechanism of these reactions, but it seems likely that the patient experienced an immediate reaction (type I hypersensitivity; Figure 7) to oral vitamin B5 after a previous contact allergy to panthenol.

The IgE-mediated nature of the reaction also appears to be confirmed by the case reported by Schalock et al. [80]. A 53-year-old woman experienced facial swelling, erythema, and pruritus, accompanied by diffuse itching of the trunk. Symptoms appeared within one minute of applying a new hair conditioner containing panthenol and gradually subsided within an hour after washing it off. Previously, the woman had experienced itchy scalp after hairdresser visits. During the diagnostic process, she underwent epidermal patch testing with the conditioner (“as is”) and with panthenol (30% PET), yielding negative results. Subsequently, skin prick tests were performed with the same sources, which were positive (2 min after pricking). These results, both from the skin and epidermal tests, suggest an IgE-mediated allergy to panthenol [80].

Each of the proposed mechanisms of panthenol hypersensitivity is plausible. The simultaneous occurrence of more than one pathogenetic pathway of vitamin B5 hypersensitivity cannot be ruled out. The occurrence of panthenol hypersensitivity likely depends on the patient’s immune status, metabolic function, the presence of other clinical conditions (primarily affecting the skin at the site of exposure), environmental conditions, the duration, frequency, and intensity of exposure to pantothenic acid, and the route of administration.

### 4.4. Pantothenic Acid Degradation Products in the Context of Hypersensitivity to Vitamin B5

Vitamin B5 is most stable at a neutral pH, but is very sensitive to moisture, heat (above 70 °C), and extreme pH (strongly acidic or strongly alkaline). In cosmetics, it is most often used in the form of calcium pantothenate or dexpanthenol, which are the forms least sensitive to environmental conditions and storage [4,7,93]. Under unfavorable environmental conditions (high or low pH, high temperature, sunlight, high humidity) or as a result of long-term storage, pantothenates contained in cosmetics degrade [94]. The degradation products of pantothenic acid and its derivatives, such as pantolactone and β-alanine, are also the main substrates for the industrial production of calcium pantothenate [7,95]. Ready-made commercial cosmetics may contain small amounts of these substrates, which are both post-production contamination and the result of degradation occurring during storage and use [7]. According to available data, it is estimated that pantolactone may be a contaminant (~1%) of cosmetics containing vitamin B5 [96,97]. Vitamin B5 can also be degraded by bacteria [98], so the degradation of pantothenic acid and its derivatives in cosmetics may be caused by microbiological contamination resulting from product contamination during use. It is worth noting that all of the above-mentioned adverse conditions, both physical, chemical, and microbiological, occur on skin, especially when covered in sweat and under high temperatures (e.g., sunny days, fever). Therefore, it is likely that the degradation of pantothenates to pantolactone and β-alanine may occur immediately after application of the cosmetic, directly to the skin [78]. Blanchard et al. [78] presented two cases of patients who developed a hypersensitivity reaction to cosmetics containing panthenol, in which the diagnostics (epidermal patch tests) proved that pantolactone was the main or only factor responsible for the allergic reaction. Panthenol allergy, although rare, seems to be a complex problem where it is impossible to exclude the involvement of a whole group of chemically related substances triggering this reaction.

## 5. Current Diagnostic Options for Vitamin B5 Hypersensitivity

The undefined and ambiguous mechanism of panthenol allergy poses a challenge in selecting an effective diagnostic strategy for suspected panthenol hypersensitivity. Much evidence suggests a late, contact hypersensitivity reaction to vitamin B5 (type IV); therefore, patch testing with the suspected allergen/hapten (panthenol) appears to be an appropriate diagnostic option. It is worth noting that panthenol (5% PET, P-042) is a component of the C-1000 cosmetic series (Chemotechnique Diagnostic, Vellinge, Sweden). This allergen series is intended for in vivo diagnostics and contains chemicals and excipients most commonly used as active substances or formulation ingredients in various cosmetics and is the only diagnostic allergen/hapten series containing panthenol. Vitamin B5 is not a consistent component of most routinely used contact allergen series. Furthermore, standard panthenol concentrations and vehicles for patch testing have not yet been validated [8].

**Figure 8:**
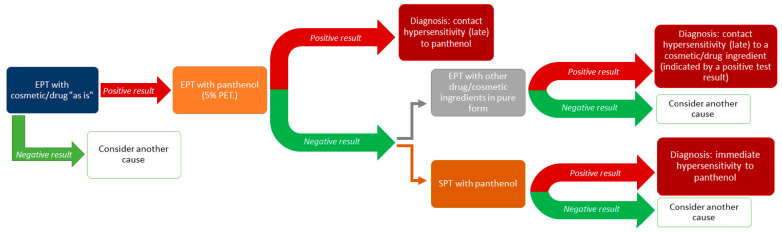
Example diagnostic strategy for additional tests in the diagnosis of panthenol hypersensitivity (author’s own figure).

The lack of standardized diagnostic tools combined with a rare allergen (hapten) causes significant difficulties in diagnosing panthenol hypersensitivity. In the diagnosis of rare allergens (including drugs and cosmetics) or substances for which there are no validated diagnostic tests, skin tests (prick or epidermal) are most often used with products indicated by the patient as a potential cause of allergy (“as is”) or with individual ingredients of this product in a pure state [99]. A similar diagnostic approach can be used to diagnose suspected panthenol hypersensitivity (Figure 8).

In cases of allergy to rare allergens/haptens found in cosmetics or drugs, this strategy is usually effective [100]. Analysis of reported clinical cases of hypersensitivity to panthenol [83] shows that in patients who experienced such a reaction, patch testing showed skin reactivity to the cosmetic (drug) used “as is,” and positive patch test results with 5% panthenol in petrolatum (5% PET) [83] or skin prick tests with panthenol [80] indicated that panthenol was the only reactive ingredient of the cosmetic (drug) that caused the hypersensitivity reaction. It also seems appropriate to take into account the degradation products of panthenol and substrates used for the industrial synthesis of vitamin B5 in the diagnosis of hypersensitivity to pantothenic acid and its derivatives [78].

## 6. Conclusions and Summary

Vitamin B5 (pantothenic acid and its derivatives) is a water-soluble B vitamin. It is a component of enzymatic systems (including CoA) crucial for cellular energy metabolism and involved in the synthesis of neurotransmitters, steroid hormones, and hemoglobin. Vitamin B5 plays an important role in maintaining the proper condition of the skin and mucous membranes, supporting their regeneration, has anti-inflammatory properties, and supports repair processes (e.g., wound healing) [101].

Pantothenic acid and its derivatives are also substances generally considered safe and non-allergenic, which, combined with their recognized skin protective and regenerative properties, makes them a common ingredient in hypoallergenic cosmetics and regenerative drugs and parapharmaceuticals that soothe irritations of the skin and mucous membranes and support the treatment of local inflammations [7,83].

According to the Expert Panel for Cosmetic Ingredient Safety (CIR) and the Scientific Committee on Consumer Safety (SCCS), panthenol, pantothenic acid, and 5 of its derivatives (panthenyl ethyl ether, panthenyl ethyl ether acetate, panthenyl triacetate, calcium pantothenate, sodium pantothenate) used in cosmetics and personal care products are generally considered safe under current use practices [7]. Cosmetics, including skin creams and serums containing up to 5% panthenol, are generally well tolerated; however, it is worth noting that no upper limit for the concentration of panthenol or its derivatives in cosmetics has been established [7]. The regulatory position emphasizes that these compounds, when used in compliant cosmetic formulas and as intended, pose a low risk to consumers [7].

Although contact allergy to panthenol is considered to be extremely rare, published data indicate an increase in the number of positive reactions, probably due to increased exposure to this ingredient in personal care products, especially so-called hypoallergenic cosmetics and cosmetics specifically intended for atopic, damaged, and inflamed skin [52,83] and in polyallergic people [77,102]. This phenomenon is probably, paradoxically, the result of the increased use of panthenol in cosmetics intended for use by people predisposed to developing allergies (e.g., atopic people) [52,83].

Diagnosing hypersensitivity to panthenol, as an allergy to a rare allergen (hapten), poses a significant diagnostic challenge both because it is not usually considered as the cause of the reaction at the initial stage of diagnosis and because of the unavailability of standard additional tests that would allow for an effective diagnosis [103,104].

Due to the existing methodological difficulties, it seems that a good diagnostic strategy in cases of suspected hypersensitivity to locally applied drugs or cosmetics is epidermal patch testing with the preparations used by the patient “as is” as a potential cause of the reaction and, in the case of positive results, extending the patch testing to individual ingredients of the cosmetic/drug in pure form. Optionally, if clinical data indicate type I reactions, skin prick testing with the suspected allergen/hapten contained in the cosmetic/drug (e.g., panthenol) may be considered [83,105,106].

In the context of the growing problem of hypersensitivity to panthenol [58,59], it is worth considering including panthenol in the basic or extended series of allergens/haptens [79]. This seems particularly justified if the diagnosis is performed in a patient with polyvalent allergy, a patient with various types of inflammatory skin lesions, or other skin conditions that predispose to allergy to topical agents, especially if the cosmetics the patient normally uses are hypoallergenic products intended for sensitive or atopic skin [83].

## Figures and Tables

**Table 1:** The function and importance of Coenzyme A (CoA) and Acyl Carrier Protein (ACP) in metabolic processes [2,6,9,25].

Carbohydrate-related an oxidative-related metabolism: Citric acid cycle transfer reactions Acetylation of sugars to build cell structure
Lipid-related metabolism: Phospholipid biosynthesis to cell membrane formation and regulation of cell membrane fluidity Isoprenoid biosynthesis to cholesterol and bile salt production Steroid and steroid hormone biosynthesis Fatty acid elongation, triacylglycerol synthesis and fatty acid Energy storage
Protein-related metabolism: Protein acetylation (changes in protein conformation, activation of peptide hormones, activation of enzymes, regulation of gene transcription) Protein acylation and prenylation (regulation of activation and bioavailability of hormones and transcription factors)

**Table 2:** Symptoms of vitamin B5 deficiency [9,27].

Neurological symptoms	Numbness, burning and tingling in the feet (“burning feet syndrome”) Muscle spasms and tremors and/or impaired muscle coordination Tingling or pins and needles (paresthesia)
Gastrointestinal symptoms	Decreased or loss of appetite and digestive problems Abdominal pain (stomach and intestinal cramps) Nausea and vomiting
Behavioral and psychological symptoms	General (chronic) fatigue and weakness Insomnia and/or sleep disturbances Irritability and anxiety Depression and low mood Headache
Symptoms affecting the skin, mucous membranes and hair	Inflammation, peeling and cracking of the skin (especially around the corners of the mouth and eyes) Keratosis epidermidis Acne Mucous membrane erosions (e.g., mouth ulcers) Premature graying of hair Slowed hair growth and excessive hair loss Brittle nails
Immune system and blood	Weakened immunity and increased susceptibility to infections (especially of the upper respiratory tract) Anemia
Other symptoms	Weight loss Increased blood pressure

**Table 3:** Recommended daily intake of vitamin B5 according to various scientific societies [30,31,32,33,34,35].

	DH	SCF	IOM	Afssa	WHO/FAO	D-A-CH
	(1991) [30]	(1993) [31]	(1998) [32]	(2001) [33]	(2004) [34]	(2013) [35]
Age (m.)			7–12	0–12	7–12	4 - < 12
AI (mg/day)			1.8	2	1.8	3
Age (y.)			1–3	1–3	1–3	1 - < 4
AI (mg/day)			2	2.5	2	4
Age (y.)			4–8	4–6	4–6	4 - < 7
AI (mg/day)			3	3	3	4
Age (y.)			9–13	7–9	7–9	7 - < 10
AI (mg/day)			4	3.5	4	5
Age (y.)			14–18	10–12	10–18	10 - < 13
AI (mg/day)			5	4	5	5
Age (y.)				13–15		13 - < 19
AI (mg/day)				4.5		6
Age (y.)				16–19		
AI (mg/day)				5		
Age (y.)	≥19	≥19	≥19	≥19	≥19	≥19
AI (mg/day)	6	5	5	5	3–12	3–7
Pregnancy						
AI (mg/day)	3–7	3–12	5	5	5	6
Lactation						
AI (mg/day)	3–7	3–12	7	7	7	6

Legend: m.—months; y.—years; AI—Adequate Intake; DH—Department of Health; SCF—Scientific Committee for Food; IOM—US Institute of Medicine; Afssa—Agence Française de Sécurité Sanitaire des Aliments (French Agency for Food Safety); WHO—World Health Organization; FAO—Food and Agriculture Organization; D-A-CH—Deutschland–Austria–Confoederatio Helvetica.

**Table 4:** Average vitamin B5 content in various foods [39].

Food	mg Per Serving	Daily Value [%]
Beef liver, boiled (≈85 g)	8.3	166
Shitake mushrooms, cooked, pieces (≈72.5 g)	2.6	52
Sunflower seeds (≈30 g)	2.4	48
Chicken, breast meat, skinless, roasted (≈85 g)	1.3	26
Tuna, fresh, cooked (≈85 g)	1.2	24
Avocado, raw (half a fruit; ≈77 g)	1.0	2.0
Milk; 2% fat (250 mL)	0.9	18
Mushrooms, white, sliced, stir fried (≈35 g)	0.8	16
Potatoes, russet, flesh and skin, baked (150–185 g)	0.7	14
Egg, hard boiled (1 large; 63–73 g)	0.7	14
Greek yogurt, vanilla, nonfat (≈150 g)	0.6	12
Ground beef, 85% lean meat, broiled (≈85 g)	0.6	12
Peanuts, roasted in oil (63–73 g)	0.5	10
Broccoli, boiled (78–92 g)	0.5	10
Chickpeas, cooked or canned (85–120 g)	0.4	8
Rice, brown, medium grain, cooked (≈100 g)	0.4	8
Oats, regular and quick, cooked with water (≈117 g)	0.4	8
Cheese, cheddar (≈42.5 g)	0.2	4
Carrots, raw (60–65 g)	0.2	4
Cabbage, boiled (≈55 g)	0.1	2
Clementine, raw (1 fruit without skin; 65–90 g)	0.1	2
Tomatoes, raw, chopped or sliced (75 to 100 g)	0.1	2

## Data Availability

No new data were created or analyzed in this study.
